# Glutathione Peroxidase 4 in Blunt Snout Bream (*Megalobrama amblycephala*) Regulates Ferroptosis and Inflammation in Response to *Aeromonas hydrophila* Infection

**DOI:** 10.3390/cimb47050326

**Published:** 2025-05-02

**Authors:** Miao He, Huanling Wang, Hong Liu

**Affiliations:** 1 Key Lab of Freshwater Animal Breeding, Ministry of Agriculture and Rural Affair/Key Lab of Agricultural Animal Genetics, Breeding and Reproduction of Ministry of Education, College of Fisheries, Huazhong Agricultural University, Wuhan 430070, China; halihe@icloud.com (M.H.); huanlingok@mail.hzau.edu.cn (H.W.); 2 Engineering Research Center of Green Development for Conventional Aquatic Biological Industry in the Yangtze River Economic Belt, Ministry of Education, Wuhan 430070, China

**Keywords:** *Megalobrama amblycephala*, *Aeromonas hydrophila*, *gpx4*, ferroptosis, inflammation

## Abstract

Glutathione peroxidase 4 (GPX4) plays a crucial role in regulating lipid peroxidation and is associated with infection and inflammation, particularly in terms of its effects on inflammatory cytokines and ferroptosis. This study aimed to investigate the regulatory effects of Gpx4 on the inflammatory response and ferroptosis in blunt snout bream (*Megalobrama amblycephala*), a significant freshwater fish species in China, after *Aeromonas hydrophila* infection. Using a bioinformatics analysis, we discovered that Gpx4 has a conserved protein structure and high amino acid identity in various carp species, indicating functional conservation across species and through involution. RT-qPCR analysis revealed that *gpx4* mRNA increased after the neuroembryonic stage during early development and was particularly highly expressed in the liver of healthy adult fish. Upon *A. hydrophila* infection, *gpx4* expression decreased significantly and rapidly in the liver. In L8824 cells, overexpression of *gpx4* suppressed inflammatory cytokines and inhibited ferroptosis in response to both *A. hydrophila* infection and induction of ferroptosis-inducer RSL3. These findings highlight the regulatory role of Gpx4 in cellular ferroptosis and inflammation, providing insights into the complex mechanisms of disease resistance and potentially aiding in the development of strategies for disease control in fish.

## 1. Introduction

Recent studies have revealed that *Aeromonas hydrophila*, a member of the *Aeromonas* genus in the Vibrionaceae family [1], can cause health issues and is considered to be a significant public health risk factor [2]. An epidemic outbreak of bacterial septicemia, primarily caused by *A. hydrophila*, particularly affects carp species such as silver carp (*Hypophthalmichthys molitrix*), bighead carp (*Aristichys nobilis*), common carp (*Cyprinus carpio*), and blunt snout bream (*Megalobrama amblycephala*) [3,4]. Blunt snout bream, a significant freshwater species in China, is frequently exposed to diseases due to its dense population and diminished genetic quality [5,6]. The infection of *A. hydrophila* can lead to severe symptoms including body bleeding, intestinal hemorrhaging, liver whitening, abdominal water accumulation, and potentially high mortality rates in blunt snout bream [7]. The extensive distribution and high infectivity of *A. hydrophila* impose a significant financial burden on the aquaculture of blunt snout bream. While the use of potent antibiotics can effectively manage the condition, the possibility of recurring outbreaks remains if prolonged usage results in heightened resistance [8]. Therefore, understanding the mechanisms of antibacterial response and enhancing the disease resistance of blunt snout bream is a more fundamental approach to addressing the pathogenicity of *A. hydrophila* in aquaculture [9].

Ferroptosis is a recently discovered form of programmed cell death that is heavily dependent on iron accumulation [10]. Excess iron catalyzes the lipid peroxidation process in membranes, ultimately leading to the accumulation of lipid peroxides to lethal levels, causing ferroptosis. The present assessment of ferroptosis includes three key markers: malondialdehyde (MDA), glutathione (GSH), and glutathione peroxidase 4 (GPX4). MDA, a byproduct of lipid peroxidation, is elevated during ferroptosis [11]. GSH exhibits strong reductive properties and can convert harmful lipid hydroperoxides generated by the cellular metabolism into harmless lipid alcohols, which is essential in influencing the occurrence of ferroptosis in cells [12,13]. GPX4, an enzyme isolated in 1982 [14], can effectively control the iron-catalyzed lipid peroxidation process in membranes, preventing the accumulation of lipid peroxides to lethal levels that cause ferroptosis. In addition to excessive iron accumulation, inhibition of GPX4 is another primary signal that can initiate ferroptosis [15]. GPX4 and GSH can inhibit ferroptosis [16], whereas their deficiency leads to the accumulation of cytotoxic reactive oxygen species (ROS), resulting in cellular ferroptosis [17].

Research on ferroptosis in inflammatory disorders focuses on modulating associated components to manipulate cellular responses and manage intracellular ferroptosis levels [18,19]. A previous study indicated a strong association between inflammation and ferroptosis, mediated by chemokines produced by renal ferrocytes [20]. GPX4, which acts as a central regulator in ferroptosis, has been shown to mitigate inflammation by alleviating oxidative stress, and augmenting its activity may represent a feasible approach for managing inflammation [19]. The activation of ferroptosis correlates with tissue damage in hosts caused by bacterial infections. Significantly, the conditional depletion of *Gpx4* in bone marrow cells hastens systemic inflammatory responses and multi-organ failure in a mouse (*Mus musculus*) model of polymicrobial sepsis induced by cecum ligation and puncture. Previous studies have shown that activating GPX4 is beneficial in mitigating inflammation [21,22,23,24]. These studies collectively indicate a significant regulatory function of GPX4 in preventing inflammatory and autoimmune disorders.

Numerous studies have shown that *GPX4* is downregulated during the inflammatory response. In the case of bacterial infection, *GPX4* expression in cells, particularly in macrophages, inhibits the activation of inflammasome [25]. Overexpression of *GPX4* prevents ferroptosis and reduces neuroinflammation by blocking the chemokine-driven recruitment of peripheral macrophages [26]. Another study has shown that the overexpression of *GPX4* reduces the bacterial load in the lungs and spleens of mice [27]. The functional investigation of *GPX4* has made significant progress, revealing its crucial involvement in the mitigation and management of inflammation in model organisms such as mice and zebrafish (*Danio rerio*). However, few studies have been conducted on *Gpx4* in economically important fish species such as blunt snout bream.

Up to date, several studies have investigated the role of the *gpx4* gene in fish immunity. For instance, one study examined the mechanisms of iron homeostasis and the GSH-GPX4 antioxidant system in response to *A. hydrophila* using grass carp (*Ctenopharyngodon idella*) spleen macrophages as a model [28]. Another study revealed that exposure to microplastics caused a downregulation of *gpx4*, leading to an intestinal inflammatory response in carp [29]. In a study examining the environmental effects on inflammation in fish, prolonged ammonia stress was found to led to alterations in *gpx4* expression and antioxidant enzyme systems, resulting in sustained oxidative stress and enhanced inflammatory responses, and ultimately causing brain tissue damage in yellow catfish (*Pelteobagrus fulvidraco*) [30]. However, there is currently no report on the role of the *gpx4* gene in bacterial inflammation in blunt snout bream.

The aim of this study is to explore the regulatory role of the *gpx4* gene in blunt snout bream in ferroptosis and inflammatory responses after *A. hydrophila* infection. Here, we characterized the *gpx4* gene in blunt snout bream, examined its expression patterns under different conditions, and measured ferroptosis markers such as MDA levels, the ratio of GSH to oxidized glutathione (GSSG), and reactive oxygen species (ROS) in cell models with *gpx4* overexpression after ferroptosis induction/bacterial infection in vivo. The findings shed light on the complex regulatory mechanisms of fish *gpx4* in ferroptosis and inflammatory responses triggered by bacterial infection, and provide a scientific foundation for addressing the problem of *A. hydrophila* resistance in aquaculture.

## 2. Material and Methods

### 2.1. Phylogenetic Analysis, Homologous Alignment, and Protein Structure

The amino acid sequences of Gpx4 from fish, amphibians, and mammals were retrieved from the NCBI database, with accession numbers listed in Table S1. These Gpx4 sequences were aligned using the default parameters of Clustal X2, and the Gpx4 phylogenetic tree was constructed using the Neighbor-Joining (NJ) method and the Dayhoff model in MEGA 11. The concordance of the Gpx4 amino acid sequences was examined utilizing the online tool available at http://www.bio-soft.net/sms/index.html accessed on 20 March 2024. The tertiary structure of Gpx4 from zebrafish, grass carp, common carp, and blunt snout bream were constructed using SWISS-MODEL (https://swissmodel.expasy.org/interactive) accessed on 13 September 2024 [31].

### 2.2. Bacterial Challenge and Sample Collection

The animal experiments were approved by the Institutional Animal Care and Use Committee (IACUC) of Huazhong Agricultural University (HZAU), Hubei, China (HZAUFI-2022-0024).

Adult blunt snout breams (500 ± 20 g) were obtained from a recirculating aquaculture system at the Aquatic Experimental Base of Huazhong Agricultural University (HZAU), Wuhan, China. Ten types of tissues, including the liver, spleen, kidney, intestine, gill, heart, muscle, brain, blood, and head kidney, from nine adult blunt snout breams were collected. Three pairs of two-year-old blunt snout bream parents were artificially inseminated, and the fertilized eggs were incubated in incubation buckets at an average water temperature of approximately 28 °C. Embryos at various developmental stages were collected and examined under a dissecting microscope. By observing embryonic development, samples were collected from twelve stages of blunt snout bream embryos, including the zygote, 2-cell, 8-cell, 32-cell, blastocyst, gastrula, neuroembryonic, eye asc, muscle effect, heartbeat effect, efferent, intestinal, and at 2 and 4 days post hatching.

All blunt snout bream juveniles (25 ± 5 g) used in the infection experiment were obtained from a fish culture farm in Honghu, Hubei, and were temporarily raised in a circular aquaculture system at the Aquatic Experiment Base of HZAU at approximately 28 °C. The experimental fish were fed pellet feed twice daily for two weeks prior to treatment. The strain of *A. hydrophila* used in this study was stored in our laboratory. Based on previous research and preliminary experiments, the final concentration of *A. hydrophila* was determined to be 6 × 10^6^ CFU/mL [32]. The experimental and control groups were intraperitoneally injected with 0.1 mL of *A. hydrophila*, respectively. Liver tissues were collected at the following time points: 0, 4, 12, 24, 48, and 120 h post injection (hpi). Nine fish were collected from each group at each time point, and every three fish were combined to create a biological replicate, resulting in a total of three replicates. All fish were anesthetized with MS-222 prior to handling. The tissue samples were taken on an ice tray, rapidly frozen using liquid nitrogen, and subsequently stored at −80 °C in an ultra-low-temperature freezer.

### 2.3. Plasmid Construction

All enzymes utilized in the cloning procedures were obtained from Thermo Fisher Scientific, Waltham, MA, USA. To construct the overexpression plasmid (pCMV-*gpx4*-N-Flag), the *gpx4* sequence from blunt snout bream were amplified via PCR using gene-specific primers that incorporated the restriction endonuclease sites Xho I and Hind III (Table S2). The pCMV-N-Flag vector (Beyotime) was employed for cloning after the sequences underwent digestion with restriction enzymes.

### 2.4. Cell Culture and Transfection

Grass carp liver cell lines (L8824 cells) stored in our laboratory were cultured in M199 medium supplemented with 100 μg/mL streptomycin, 10% fetal bovine serum, and 100 U/mL penicillin at 28 °C in a 5% CO_2_ atmosphere within a constant temperature incubator. The cells were passaged every two days. For the subsequent experiments, only cells in the logarithmic growth phase were utilized. Transient transfection of L8824 cells was performed using Lipofectamine 2000 (Invitrogen, Thermo Fisher Scientific, USA) on 96-well, 24-well, 12-well, 6-well, and 60 mm plates for functional analysis. The L8824 cells were transfected with either pCMV-*gpx4*-N-Flag or pCMV-N-Flag in the culture medium, and the cells were subsequently harvested for further assays.

### 2.5. Gene Overexpresstion

To overexpress the *gpx4* gene, we engineered the open reading frame of the blunt snout bream *gpx4* into the pCMV-N-flag vector (Beyotime Biotech, Shanghai, China). Subsequently, the plasmid was transfected into the cells using the plasmid transfection reagent Lipofectamine 2000. Finally, real-time quantitative PCR was used to detect the expression level of *gpx4* in cells to confirm whether it was overexpressed.

### 2.6. Bacterial Infection and Ferroptosis-Inducer RSL3 Treatment

*A. hydrophila* was cultured in LB medium at 28 °C until the optical density at 600 nm (OD600) reached approximately 0.8. The bacteria were then washed thrice with phosphate-buffered saline (PBS) (Gibco, Thermo Fisher Scientific, USA) and then re-suspended in PBS to achieve a final concentration of 1 × 10^3^ CFU/mL. Subsequently, the bacteria were added to the cell culture medium to obtain a concentration of 1 × 10^2^ CFU/mL, and the cells were incubated for 2 h. After removing the medium containing the bacteria, the cells were washed twice with PBS (In order to prevent bacteria that have not entered the cells from interfering with the experiment, the cells were washed twice with PBS to wash away the bacteria remaining in the culture medium that have not entered the cells.) and then transferred to fresh medium for an additional 2 h incubation.

Cells were cultured until 4 h prior to sample collection. The old medium was removed, and the cells were washed twice with PBS. RAS-selective lethal 3 (RSL3) (Glpbio, Montclair, CA, USA) was added to the fresh medium at a final concentration of 3 μM/mL. After removing the remaining PBS, the fresh medium containing RSL3 was added and the cells were incubated for 4 h.

### 2.7. RNA Extraction and Real-Time Quantitative PCR (RT-qPCR)

Total RNA was extracted from tissue and cell samples using Trizol reagent (Invitrogen, Thermo Fisher Scientific, USA) per the manufacturer’s instructions. RNA quality and integrity were assessed using agarose gel electrophoresis and measured with a NanoDrop 2000 (Thermo Fisher Scientific, USA). Complementary DNA (cDNA) was synthesized using an RNA reverse transcription kit (Novican). The RT-qPCR reaction system: SYBR Premix Ex Taq (Takara Bio, Beijing, China) 10 μL, cDNA template 1 μL, primers (both upstream and downstream) 0.8 μL, and RNase-free ddH_2_O 8.2 μL. The 18S rRNA served as the reference standard for mRNA detection in the tissues of blunt snout bream [32,33]. β-actin was used as the internal reference for mRNA expression in tissues and L8824 cells. Gene expression at different time points following *M. amblycephala* infection was compared with the 0 h control. All assays were performed in triplicate, and the primers used for RT-qPCR analysis are listed in Table S2.

### 2.8. CCK-8 Cell Proliferation Assay

A 4 × 10^3^ L8824 cells suspension of 100 μL was pipetted into each well of a 96-well plate and incubated for 12 h in a 5% CO_2_ incubator at 28 °C until them attaching to the bottom of the wells. Transfection, bacterial immersion, and RSL3 treatments were performed separately. L8824 cell wells without any treatment were used as blank controls, and all groups were further cultured in a 5% CO_2_ incubator at 28 °C for 24 h. Subsequently, 100 μL of media containing 10% CCK-8 (Biosharp, Beijing, China) was added to each well and incubated for 2 h in the dark. An enzyme-labeled instrument (SPARK, Tecan Trading AG, Männedorf, Switzerland) was utilized to measure the absorbance of each well at 450 nm. These tests were conducted in triplicate.

### 2.9. Measurement of MDA Content, GSH/GSSG Ratio, and ROS Generation

L8824 cells were plated at a density of 5 × 10^6^ cells per plate in a 60 mm plate and incubated for 12 h. Following treatment, MDA levels were measured using a Lipid Peroxidation (MDA) assay kit (Elabscience Biotechnology Co., Wuhan, China) and normalized to protein content using the BCA method (Elabscience Biotechnology Co., Wuhan, China). All experiments were conducted in triplicate.

Cells were seeded at a density of 2 × 10^5^ per well in a 6-well plate and incubated for 12 h. Following treatment, the cells were laid and analyzed for glutathione redox status (GSH/GSSG) using a GSH/GSSG kit (Elabscience Biotechnology Co., Wuhan, China). The GSH:GSSG ratio was calculated using the formula (GSH − 2GSSG)/GSSG, with triplicates measurements performed.

L8824 cells were plated at a density of 1 × 10^5^ cells per well in a 12-well plate and incubated for 12 h until being laid. ROS generation was quantified using the Reactive Oxygen Species Assay Kit (Elabscience Biotechnology Co., Wuhan, China) and expressed as arbitrary units (AU) of DCF fluorescence measured using an enzyme-labeled instrument (PE EnVision, Denver, CO, USA). All experiments were performed in triplicate.

### 2.10. Statistical Analysis

The relative gene expression data were analyzed using the 2^−ΔΔCt^ method. A significance value of *p* < 0.05 was considered statistically significant, while a significance value of *p* < 0.01 was considered extremely statistically significant. The results of the RT-qPCR were presented as mean ± standard deviation (SD) in the statistical analysis. To assess the significance of the differences among two or more groups, Student’s *t*-test or a one-way analysis of variance (ANOVA) was employed in GraphPad Prism 8.0.

## 3. Results

### 3.1. Characterization of Gpx4

**Figure 1:**
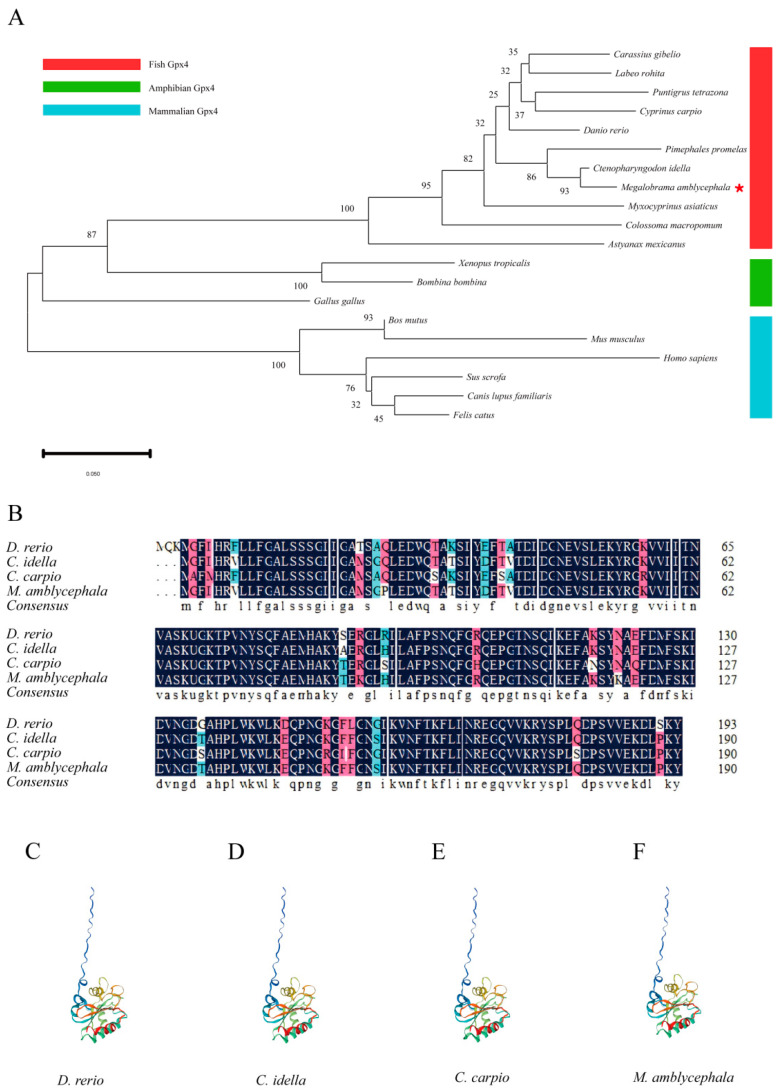
Characterization of vertebrates Gpx4. (**A**) Phylogenetic tree of vertebrates Gpx4 (the red pentagrams are labeled as the target specie of the study). (**B**) Multiple sequence alignment of Gpx4 amino acid of four cyprinid fish species. Same sequences are shown in navy blue, with conservative and semi-conservative mutations depicted in pink and blue, respectively. (**C**–**F**) Schematic diagram of the tertiary structure of Gpx4 protein of four cyprinid fish species.

The Gpx4 proteins from selected species were classified into three distinct evolutionary lineages within the evolutionary tree (Figure 1A), encompassing fish, amphibians, and mammals. Moreover, there was a significant divergence between the mammals and amphibians Gpx4, which ultimately converged with the Gpx4 found in bony fish. Additionally, we conducted a comprehensive analysis of the amino acid sequences and predicted the tertiary structures of Gpx4 in four different cyprinid fish species (Figure 1B). Compared to mammals, the amino acid sequences of blunt snout bream Gpx4 exhibited higher similarities with those of grass carp, common carp, and zebrafish, with identity percentages of 97.9%, 88.4%, and 90.2%, respectively (Table S3). These results indicate that the amino acid sequences and tertiary structures of Gpx4 are similar across all of these fish species, further supporting their high level of conservation (Figure 1C–F). Due to the lack of cell lines specifically derived from blunt snout bream tissues, the aforementioned bioinformatics analysis of the blunt snout bream *gpx4* gene has identified the L8824 cell line as a viable option for conducting cell assays pertinent to this study.

### 3.2. Expression of gpx4 During Embryonic Development, in Healthy Fish and After A. hydrophila Infection

**Figure 2:**
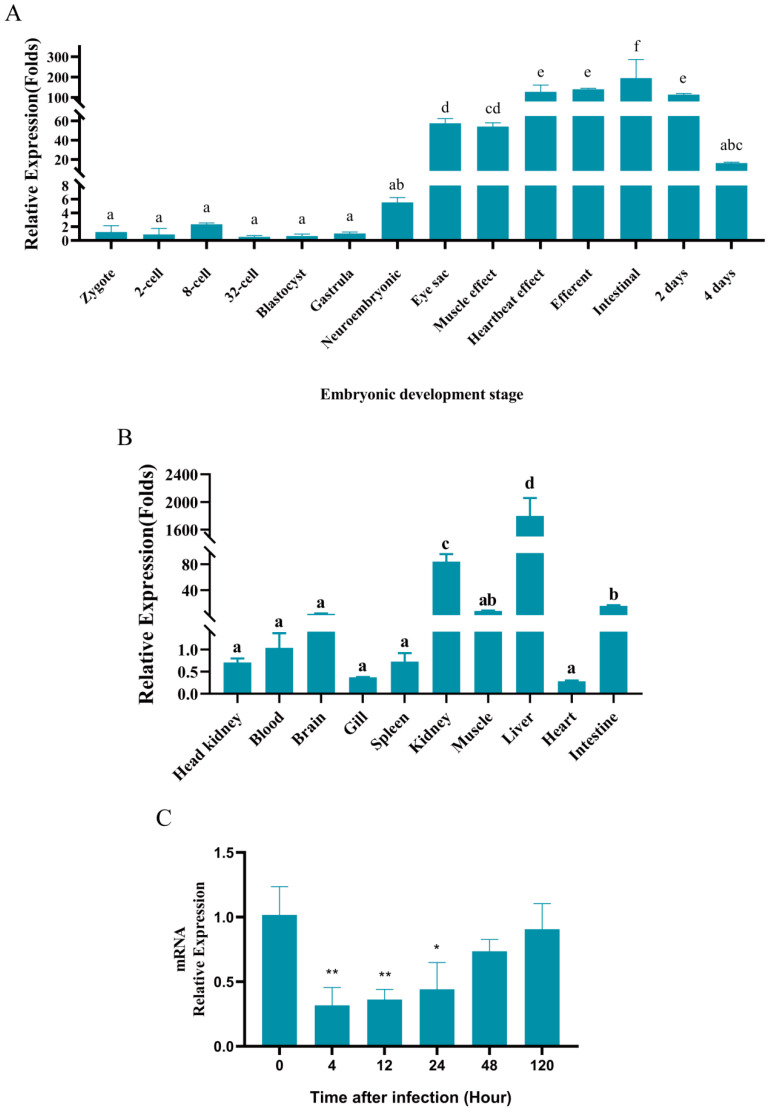
Relative expression of *gpx4*. (**A**) Expression of *gpx4* at different stages during the embryonic development of *M. amblycephala*. (**B**) Expression of *gpx4* in healthy *M. amblycephala* tissues. The letters from a to f indicate that differences in gene expression at various stages of embryonic development and across different tissues are significant (*p* < 0.05). The same letters signify no significant change. Data are described as mean ± SE (n = 3). (**C**) Expression of *gpx4* in the liver after *A. hydrophila* infection. Data are described as mean ± SE (n = 3). Statistically significant up-regulation or down-regulation of gene expression is denoted with * (*p* < 0.05), and ** (*p* < 0.01).

The levels of *gpx4* mRNA were assessed using RT-qPCR across various embryonic developmental stages and in different organs of adult fish. Our investigation revealed that the expression of the *gpx4* gene was minimal, nearly absent, during the early stages of embryonic development, from the fertilized egg stage until the gastrula stage. However, a significant increase in expression was observed starting from the formation of the neuroembryo stage, with the highest level recorded at the intestinal stage, notably higher than those observed during other stages (Figure 2A), when the membranes were exserted. In healthy adult fish, *gpx4* mRNA was particularly significantly highly expressed in the liver, with levels several hundred to several thousand times higher than in other tissues. Furthermore, the expression of GPX4 mRNA in the kidney and intestine was significantly higher than that in other tissues such as the head, kidney, blood, and spleen, etc., where the expression of GPX4 mRNA was low and the difference was not significant (Figure 2B).

The *gpx4* gene exhibited the highest expression in the liver of blunt snout bream, indicating that its primary function is in the liver. Consequently, we investigated the expression of *gpx4* in the liver tissues of blunt snout bream following *A. hydrophila* infection at various time intervals. Our observations revealed a temporal pattern in the expression of the *gpx4* gene, which is characterized by an initial decrease followed by an increase. More specifically, the expression of *gpx4* was significantly reduced at 4 hpi, reaching its minimum level at that time, before returning to baseline levels (Figure 2C).

### 3.3. gpx4 Overexpression Inhibited Expression of Inflammatory Factors

**Figure 3:**
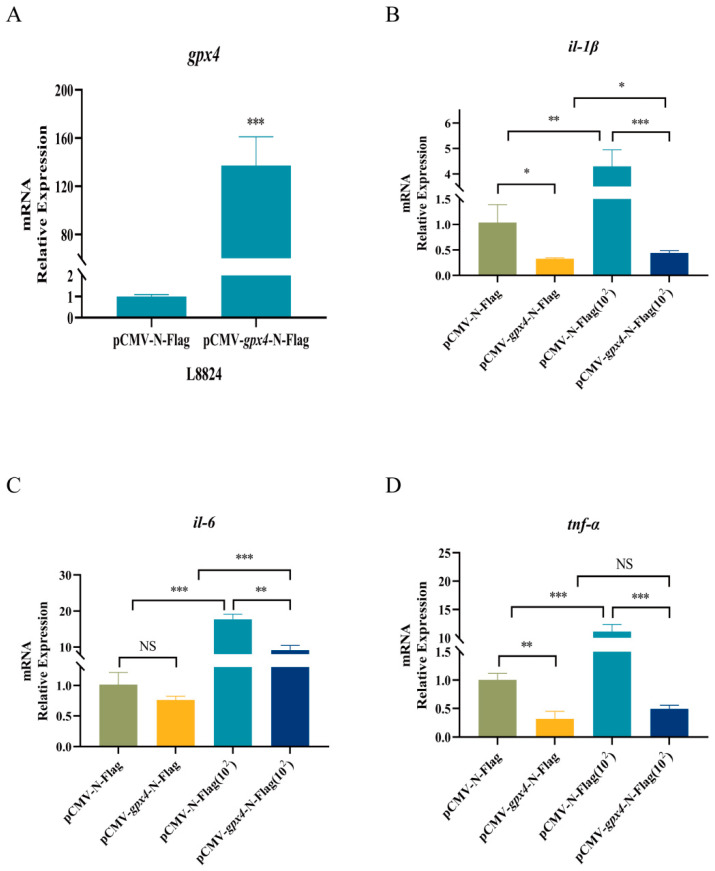
Overexpression of *gpx4* inhibited expression of inflammatory factors in L8824 cells after *A. hydrophila* infection. (**A**) Detection of the overexpression effect of *M. amblycephalagpx4* gene in L8824 cells. (**B**–**D**) Expression levels of *il-1β*, *il-6* and *tnf-α* in L8824 cells transfected with pCMV-*gpx4*-N-Flag plasmid or pCMV-N-Flag plasmid (control group). L8824 transfected cells were infiltrated with *A. hydrophila* solution (resuspended in PBS) at a concentration of 1 × 10^2^ CFU/mL for 2 h, and for the control group, the same volume of PBS was added. After treating the cells with bacteria or PBS for 2 h, the cells were washed using PBS and then continued to be incubated in the culture medium for 2 h. pCMV-*gpx4*-N-Flag(10^2^) and pCMV-N-Flag(10^2^) indicate L8824 cells treated with *A. hydrophila*. Data are described as mean ± SE (n = 3). Statistically significant up- or down-regulation of gene expression is denoted with * (*p* < 0.05), ** (*p* < 0.01), or *** (*p* < 0.001) and “NS” means no significance.

To investigate the regulation of *gpx4* on inflammatory factors, we first overexpressed the blunt snout bream *gpx4* gene by transfecting pCMV-*gpx4*-N-Flag plasmid into L8824 cells. The RT-qPCR results show that *gpx4* exhibited a significantly elevated expression level, approximately 300-fold, in L8824 cells following pCMV-*gpx4*-N-Flag transfection, suggesting that this model could be used as a *gpx4* overexpression cell model for future experiments (Figure 3A).

Subsequently, we conducted an infection experiment by incubating *A. hydrophila* with the cells. This experiment was set up with four groups: a control group with no treatment (pCMV-N-Flag), a group overexpressing *gpx4* (pCMV-*gpx4*-N-Flag), an *A. hydrophila* infection group (pCMV-N-Flag(10^2^)), and a group overexpressing *gpx4* and infected with *A. hydrophila* (pCMV-*gpx4*-N-Flag(10^2^)). The expression levels of three inflammatory factors, namely *interleukin-1 beta* (*il-1β*), *interleukin-6* (*il-6*), and *tumor necrosis factor-alpha* (*tnf-α*), were then measured using RT-qPCR. The results show that the expression levels of *il-1β* and *tnf-α* were significantly reduced in cells overexpressing *gpx4* prior to infection with *A. hydrophila*. Furthermore, the results indicate that the levels of *il-1β*, *il-6*, and *tnf-α* mRNA increased following *A. hydrophila* infection, with the most notable elevation observed in the expression of *il-6*. However, the expression levels of the three inflammatory factors were markedly reduced by the overexpression of *gpx4* in the pCMV-gpx4-N-Flag(10^2^) group compared with the pCMV-N-Flag(10^2^) group (Figure 3B–D).

### 3.4. Overexpression of gpx4 Inhibits RSL3-Induced Ferroptosis in L8824 Cells

**Figure 4:**
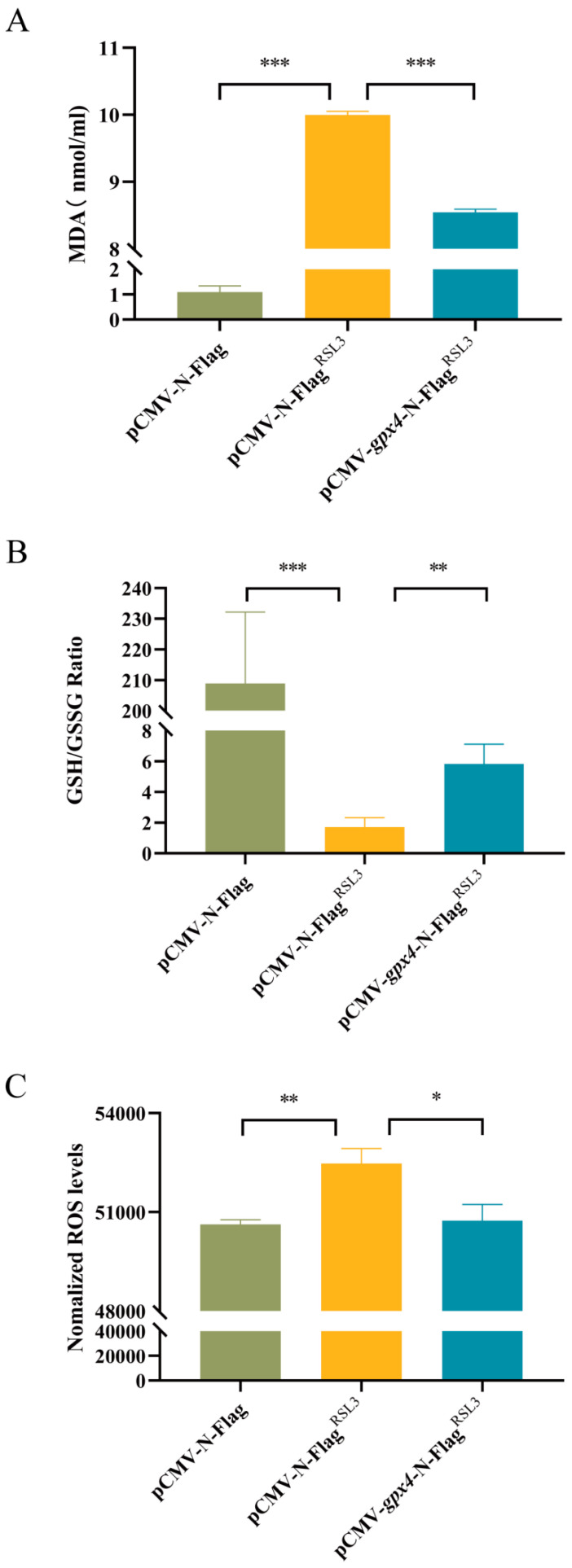
Overexpression of *gpx4* inhibited RSL3-induced ferroptosis in L8824 cells. (**A**) Changes in intracellular malondialdehyde (MDA) levels. (**B**) Changes in intracellular GSH/GSSG. (**C**) Changes in intracellular ROS. L8824 transfected cells were treated with 3 μM RSL3 for 4 h. Intracellular ROS production was analyzed and expressed as DCF fluorescence values. Data are described as mean ± SE (n = 3). Statistically significant up- or down-regulation of changes in ferroptopsis-related markers is denoted with * (*p* < 0.05), ** (*p* < 0.01), or *** (*p* < 0.001).

To investigate the impact of *gpx4* on the extent of ferroptosis, we conducted a ferroptosis induction experiment using a cell model that overexpressed the *gpx4* gene. The experiment was set up with three groups: a control group with no treatment (pCMV-N-Flag), a RSL3 induction group (pCMV-N-Flag^RSL3^), and a group overexpressing *gpx4* and induced with RSL3 (pCMV-*gpx4*-N-Flag^RSL3^). Subsequently, the ferroptosis-related parameters in the cells subsequent to the administration of the ferroptosis inducer RSL3 were assessed. The levels of MDA, the end product of lipid hydroperoxides, were significantly elevated after ferroptosis induction in the pCMV-N-Flag^RSL3^ group. Conversely, MDA levels were markedly decreased in cells with *gpx4* overexpression in the pCMV-*gpx4*-N-Flag^RSL3^ compared with the pCMV-N-Flag^RSL3^ group (Figure 4A). Ferroptosis induction significantly decreased the intracellular GSH/GSSG ratio in the pCMV-N-Flag^RSL3^ group compared with the pCMV-N-Flag group, while *gpx4* overexpression increased this ratio (Figure 4B). The results demonstrate a substantial increase in ROS content in the pCMV-N-Flag^RSL3^ group after ferroptosis induction. However, *gpx4* overexpression in the pCMV-*gpx4*-N-Flag^RSL3^ group had a notable reduction in ROS levels compared with the pCMV-N-Flag^RSL3^ group (Figure 4C).

### 3.5. gpx4 Overexpression Enhanced Cell Viability and Inhibited Ferroptosis Induced by A. hydrophila Infection

**Figure 5:**
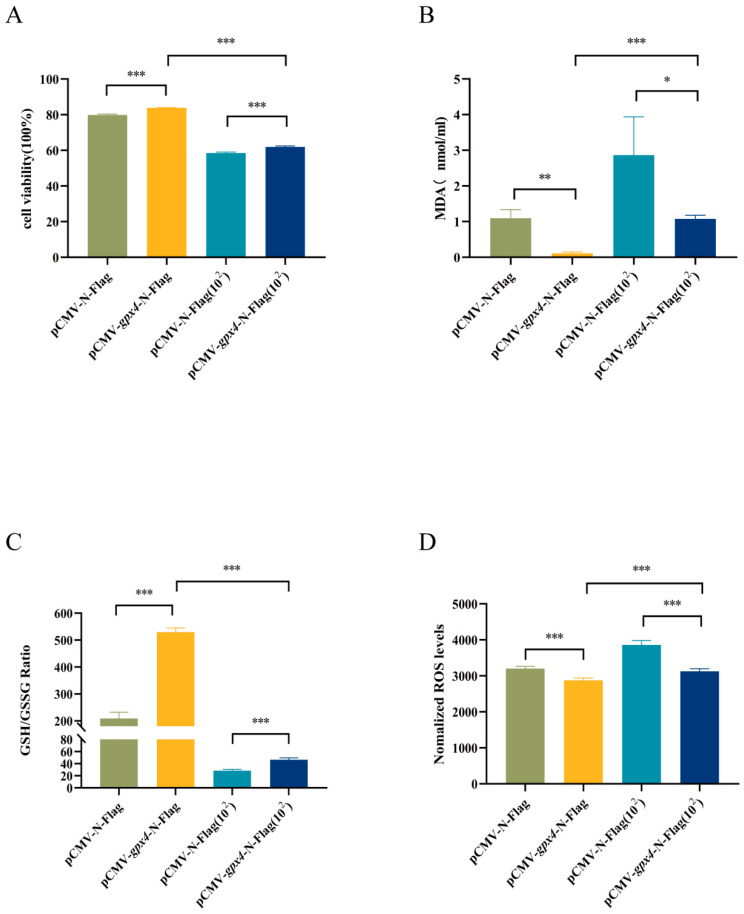
Overexpression of *gpx4* enhanced cell viability and inhibited ferroptosis induced by *A. hydrophila* infection. (**A**) Cell viability detected by cell proliferation-toxicity assay CCK-8. (**B**) Intracellular malondialdehyde (MDA) levels. (**C**) Changes in intracellular GSH/GSSG. (**D**) Changes in intracellular ROS. Intracellular ROS production was analyzed and expressed as DCF fluorescence values. L8824 transfected cells were infiltrated with *A. hydrophila* solution (resuspended in PBS) at a concentration of 1 × 10^2^ CFU/mL for 2 h, and for the control group, the same volume of PBS was added. After treating the cells with bacteria or PBS for 2 h, the cells were washed using PBS and then continued to be incubated in the culture medium for 2 h. pCMV-*gpx4*-N-Flag(10^2^) and pCMV-N-Flag(10^2^) indicates L8824 cells treated with *A. hydrophila*. Data are described as mean ± SE (n = 3). Statistically significant up- or down-regulation in the expression of genes is denoted with * (*p* < 0.05), ** (*p* < 0.01), or *** (*p* < 0.001).

To investigate the potential role of *gpx4* in cell viability and ferroptosis induced by *A. hydrophila*, an experiment was conducted with four groups: a control group with no treatment (pCMV-N-Flag), a group overexpressing *gpx4* (pCMV-*gpx4*-N-Flag), an *A. hydrophila* infection group (pCMV-N-Flag(10^2^)), and a group overexpressing *gpx4* and infected with *A. hydrophila* (pCMV-*gpx4*-N-Flag(10^2^)). Ferroptosis-related indicators, including cell viability, MDA, GSH/GSSG ratio, and ROS levels, were then examined. Following *A. hydrophila* infection, cell viability was markedly reduced. However, cell viability was significantly higher in the groups overexpressing *gpx4* (Figure 5A). After *A. hydrophila* infection, MDA levels were considerably increased, but the MDA content was significantly inhibited by the overexpression of the *gpx4* gene (Figure 5B). Additionally, *A. hydrophila* infection resulted in a marked decrease in the GSH/GSSG ratio. In contrast, overexpression of the *gpx4* gene significantly increased the GSH/GSSG ratio (Figure 5C). The results indicate a substantial increase in intracellular ROS content following *A. hydrophila* infection; however, the overexpression of the *gpx4* gene led to a significant reduction in ROS accumulation within the cells (Figure 5D).

## 4. Discussion

Our study aims to elucidate the regulatory function of the *gpx4* gene in blunt snout bream concerning ferroptosis and inflammatory responses during *A. hydrophila* infection. By characterizing the *gpx4* gene, analyzing its expression patterns, and quantifying ferroptosis indicators such as MDA, the GSH/GSSG ratio, and ROS in models involving gpx4 manipulation and bacterial challenge, we gained insights into the complex regulation of *gpx4* in fish during these processes.

First, to understand the evolutionary relationship of Gpx4 among fish, amphibians, and mammals, and the functional conservation of Gpx4 proteins in cyprinid fish including blunt snout bream, we conducted a phylogenetic analysis, amino acid sequence alignment, and tertiary structure prediction of Gpx4. A similar method was applied in the bioinformatics analysis of another gene in common carp and grass carp [34,35]. Previously reported in fish, Gpx4 possesses a unique monomer structure and plays a crucial role in the processing of certain complex lipids in the extracellular environment [36,37]. Our analysis shows that the blunt snout bream Gpx4 is most closely related to that of grass carp, with an amino acid sequence identity of 97.9%. In addition, the high similarity in the tertiary structure of the Gpx4 protein among the four cyprinid fish species suggested that they may perform similar functions.

To investigate the temporal and spatial expression patterns of *gpx4*, we examined and analyzed the relative expression levels of *gpx4* during the early development of blunt snout bream and in differential tissues in adult fish. Our findings indicate that *gpx4* is significantly expressed from the neuroembryonic stage during embryonic development, suggesting that it may play a crucial role from this developmental period onward. Furthermore, the highest expression of *gpx4* was observed in the liver of blunt snout bream. Similarly, the highest expression of *gpx4* was observed in the liver among various tissues in southern bluefin tuna [38]. Previous studies showed that liver is a key metabolic organ, and fish livers adapt to adverse conditions by altering the lipid metabolism [39,40,41]. Additionally, the liver is a crucial immune organ, exposed to antigens and endotoxins from the intestinal microbiota [42]. Its rich innate immune cell population is associated with diseases linked to intestinal immune dysregulation [43]. As an important immune organ in fish [44], it indicated that liver may have a significant role in the immunological processes of blunt snout bream.

We further assessed the changes in *gpx4* expression in the liver of blunt snout bream in response to *A. hydrophila* infection. The most significant decrease in *gpx4* expression was observed at 4 hpi, indicating that this gene in blunt snout bream is sensitive to *A. hydrophila* infection and that it may serve as a potential marker for the detection of *A. hydrophila* infection. Over time, the difference in *gpx4* expression decreased, with no significant change by 48 hpi, suggesting that the fish may adapt by increasing *gpx4* expression to reduce bacterial damage to the liver. According to previous studies, reduced GPX4 expression has been linked to several diseases. In Crohn’s disease, GPX4 activity is diminished [45], impairing its role in inhibiting lipid peroxidation and regulating ferroptosis. Furthermore, GPX4 has been shown to confer protective effects against sepsis in mice [20]. Several studies have demonstrated that there is a significant reduction in intracellular GPX4 levels following inflammation [46], which is consistent with findings observed in psoriasis [47]. Additionally, lung damage in Mtb-infected mice is correlated with decreased levels of Gpx4 [48]. Our findings underscore *gpx4* as a pivotal regulator of inflammatory responses and ferroptosis during *A. hydrophila* infection, although the molecular intricacies demand systematic investigation. Emerging evidence positions GPX4 within the Nrf2-Keap1 antioxidant axis, where Nrf2 activation transcriptionally upregulates GPX4 to curb lipid peroxidation—a relationship disrupted during pathogen challenge in blunt snout bream, potentially driving ferroptotic cascades [49,50]. Parallel mechanisms may involve compromised cysteine availability: SLC7A11, the catalytic subunit of System Xc−, sustains GPX4 activity by maintaining glutathione biosynthesis. Its suppression under oxidative duress could critically impair cellular redox balance, accelerating ferroptosis [51]. Notably, GPX4 depletion may unleash pro-ferroptotic mediators like ACSL4 and ALOX5, which actively propagate lipid peroxidation—a biochemical signature of ferroptosis [52,53]. The anti-inflammatory dimension of GPX4 likely intersects with canonical signaling pathways. Cross-regulation between Nrf2/GPX4 and NF-κB/MAPK axes could mechanistically explain reduced *il-1β* and *tnf-α* levels, as Nrf2 activation suppresses NF-κB-driven cytokine production [19,25,46]. However, these network interactions remain hypothetical in teleost models and require direct validation. Future studies should validate these interactions in blunt snout bream to elucidate the full mechanistic network. While our study demonstrates that *gpx4* overexpression attenuates inflammation and ferroptosis, the precise molecular cascades remain to be mapped. Gene editing (e.g., CRISPR/Cas9 knockout of *gpx4* or *nrf2*) and proteomic analyses could clarify these pathways. Additionally, measuring ACSL4/ALOX5 activity and NF-κB/MAPK phosphorylation in *gpx4*-manipulated models would provide deeper mechanistic insights.

The L8824 cell line served as a convenient model for probing *gpx4* functionality in *M. amblycephala*, although we acknowledge that interspecies disparities between grass carp and blunt snout bream could subtly alter cellular responses to infection or ferroptotic stimuli. While phylogenetic and structural comparisons confirm strong GPX4 conservation across cyprinids [34,35], finer-scale divergences—such as immune signaling kinetics, redox-sensing mechanisms, or GPX4 protein–protein interactions—might exist. For example, species-specific nuances in cytokine cascades or iron homeostasis could complicate the translation of our in vitro observations to *M. amblycephala* physiology. Admittedly, the use of L8824 cells stems from a persistent limitation in aquatic research: the absence of stable *M. amblycephala*-derived cell lines [54,55]. Previous work has justified this surrogate system by demonstrating functional overlap in core pathways (e.g., IL-6/STAT3 and NF-κB responses) between the two species [54,56]. Nevertheless, future studies should prioritize establishing primary hepatocyte cultures from *M. amblycephala* or developing in vivo models (e.g., CRISPR/Cas9-mediated *gpx4* knockout/knock-in systems) to validate these findings directly in the target species. Such species-matched models would not only validate our findings, but also reveal context-dependent regulatory features of GPX4 in ferroptosis and immunomodulation.

To investigate the role of blunt snout bream *gpx4* gene in ferroptosis, RSL3 was used to induce ferroptosis in L8824 cells, and ferroptosis-related indicators were measured after induction and *gpx4* overexpression. The results show that *gpx4* overexpression inhibited RSL3-induced ferroptosis in L8824 cells. RSL3 is a small molecule that induces cell death in RAS-mutant cancer cells through iron-dependent non-apoptotic mechanisms, making it a potent ferroptosis initiator [52,57,58]. RSL3 enhances ferroptosis by suppressing GPX4, an antioxidant enzyme that prevents lipid peroxidation [59]. RSL3 also inhibits LPS-induced inflammatory cytokine production in microglia and induces ferroptosis in rat embryonic cardiomyocyte (H9C2) cells [60]. Additionally, prior research has simulated ferroptosis in a hepatocyte cell line derived from zebrafish using RSL3. In this investigation, a cell model of ferroptosis was established in L8824 cells utilizing RSL3. Following the overexpression of the *gpx4* gene, alterations in markers associated with ferroptosis, such as MDA contents, GSH/GSSG ratio, and ROS levels, were observed in the RSL3-induced ferroptosis L8824 cell models. It was found that the overexpression of *gpx4* could mitigate the extent of ferroptosis induced by RSL3, indicating that the *gpx4* gene in blunt snout bream may inhibit cell ferroptosis by decreasing lipid peroxidation levels (such as MDA and ROS levels), thereby lessening the impact of *A. hydrophila* on the cells.

Key markers of ferroptosis include gpx4 expression, MDA content, the GSH/GSSG ratio, ROS levels, Fe^2+^ accumulation, and mitochondrial morphology [61,62]. Gpx4, an antioxidant enzyme that prevents lipid peroxidation and regulates ferroptosis, which is marked by free iron accumulation and oxidative stress [63]. MDA can be used to indicate lipid peroxidation, while decreased GSH, increased ROS, and heightened lipid peroxidation initiate ferroptosis [53]. A study showed that *GPX4* overexpression significantly suppressed ferroptosis in the mouse hippocampus [26]. In Alzheimer’s and Parkinson’s disease models, ferroptosis is characterized by decreased MDA and GSH, increased ROS, and elevated lipid peroxidation [64]. In erastin-treated dermatitis models, MDA levels rose in keratinocytes, with increased ROS and a reduced GSH/GSSG ratio; however, Fer-1 pretreatment reversed these effects [47]. In this study, we evaluated the impact of *gpx4* gene overexpression on a cell model infected with *A. hydrophila* using a cell viability assay. Additionally, we measured lipid peroxidation levels, including MDA content, the GSH/GSSG ratio, and ROS production, to assess the influence of *gpx4* overexpression on intracellular ferroptosis. Our findings show that overexpression of the blunt snout bream *gpx4* gene can suppress ferroptosis caused by *A. hydrophila*, reducing lipid peroxidation and improving cell viability. This suggested that the *gpx4* gene may alleviate the damage inflicted on the organism during bacterial infection by modulating ferroptosis, thereby indicating a potential role for ferroptosis in specific microbial infectious diseases.

Ferroptosis contributes to inflammation, and its inhibitors can offer anti-inflammatory effects [65]. A previous study found that elevated inflammatory markers in the colonic tissues of a mouse model of ulcerative colitis significantly decreased with Fer-1 therapy [66]. The potential role of *A. hydrophila* in regulating inflammation in blunt snout bream by inhibiting ferroptosis remains uncertain. In this study, we conducted a preliminary investigation into the roles of the blunt snout bream *gpx4* gene in inflammation and ferroptosis in vitro. The findings suggest that overexpression of the *gpx4* gene may modulate the response to bacterial infection by inducing the inflammatory cytokines and suppressing ferroptosis. However, it remains unclear whether this gene performs the same function in the liver of blunt snout bream in suppressing inflammation and ferroptosis in vivo. To solve this problem, it is necessary to further study the function of the *gpx4* gene at the protein level, or using gene editing, and explore its related signaling pathway.

## 5. Conclusions

This study elucidates the regulatory function of *gpx4* in the inflammatory response to *A. hydrophila* infection in blunt snout bream. The overexpression of *gpx4* attenuated the inflammatory reaction and ferroptosis triggered by *A. hydrophila*, indicating its crucial role in anti-inflammatory process. This study provides new insights into the regulatory network of ferroptosis in fish and new strategies for enhancing resistance to *A. hydrophila* in blunt snout bream during aquaculture.

## Data Availability

The data are contained within the article.

## Supplementary Materials

The following supporting information can be downloaded at: https://www.mdpi.com/article/10.3390/cimb47050326/s1.
