# Supplementary material for: Glutathione Peroxidase 4 in Blunt Snout Bream (Megalobrama amblycephala) Regulates Ferroptosis and Inflammation in Response to Aeromonas hydrophila Infection
Source: Curr Issues Mol Biol. 2025 May 2;47(5):326. doi: 10.3390/cimb47050326 (PMC12109816; doi:10.3390/cimb47050326)
Supplement: Supplementary file 1 [file cimb-47-00326-s001.zip › cimb-3563435-supplementary.pdf]

Table S1 Species names and GenBank numbers used in this study.

Species names	Gene/Protein names	GenBank numbers
Megalobrama amblycephala	*gpx4*	XM_048165154.1
	Gpx4	XP_048021111.1
Ctenopharyngodon idella	*gpx4*	XM_051907666.1
	Gpx4	XP_051763626.1
	il-1β	MK_942107.1
	il-6	XM_051893317.1
	tnf-α	JQ_670915.1
Carassius gibelio	Gpx4	XP_052395858.1
Labeo rohita		XP_050951370.1
Puntigrus tetrazona		XP_043079734.1
Cyprinus carpio		XP_042605780.1
Danio rerio		NP_001333466.1
Pimephales promelas		XP_039538542.1
Myxocyprinus asiaticus		XP_051550741.1
Colossoma macropomum		XP_036441876.1
Astyanax mexicamus		XP_049321249.1
Xenopus tropicalis		NP_001291701.1
Bombina bombina		XP_053558786.1
Gallus gallus		NP_001333377.1
Bos mutus	GPX4	XP_014332058.1
Mus musculus		NP_001032830.2
Homo sapiens		NP_001034936.1
Sus scrofa		NP_999572.1
Canis lupus familiaris		XP_038285274.1
Felis catus		XP_044898589.1

Table S2 Primers used in this study.

Primer name	Primer sequence（5’ -3’）	Usage
β-actin -F	TCGTCCACCGCAAATGCTTCTA	RT-qPCR
β-actin -R	CCGTCACCTTCACCGTTCCAGT	
C β-actin -F	ACCCACACCGTGCCCATCTA	RT-qPCR
C β-actin -R	CGGACAATTTCTCTTTCGGCTG	
C-*18S* rRNA-F	ATTTCCGACACGGAGAGG	RT-qPCR
C-*18S* rRNA-R	CATGGGTTTAGGATACGCTC	
gpx4-F	TTTTAGGCTCTGCTGTCG	PCR、
gpx4-R	ACTGGTTGGATGGGAAGG	RT-qPCR
C *gpx4*-F	CTGCAGGATCCAAGTGTGGT	PCR、
C *gpx4*-R	GTAGAAGGACGGGGGTCTCT	RT-qPCR
gpx4-C-F	CCCAAGCTTATGCACGCCAAGTACACTGAGA	Overexpression
gpx4-C-R	CCGCTCGAGGAGATATTTAGGCAGATCCTTTTCC	construction
il-1β-F	TCTCCTCGTCTGCTGGGTGT	RT-qPCR
il-1β-R	CAAGACCAGGTGAGGGGAAG	
il-6-F	AACCCTGGTCAACGACATCA	RT-qPCR
il-6-R	CCACCCTTCCTCTTGCTCAT	
tnf-α-F	ACCAGGACCAGGCTTTCTCT	RT-qPCR
tnf-α-R	GCATAACTGCGTGGCTCATA	

Note: The GenBank accession numbers of the nucleotide sequence used to design the primer are as follows:

gpx4 (XM_048165154.1); C *gpx4* (XM_051907666.1); *il-1β* (MK_942107.1); *il-6* (XM_051893317.1); *tnf-α* (JQ_670915.1)；β-actin (XM_048192430.1); C β-actin (XM_051886219.1); *18S* rRNA (XM_048161194.1)

Table S3 Identity percent analysis of Gpx4 amino acid sequences.

Species	*M. amblycephala*	*C. idella*	*C. carpio*	*D. rerio*	*H. sapiens*	*M. musculus*
M. amblycephala	100%					
C. idella	97.9%	100%				
C. carpio	88.4%	89.5%	100%			
D. rerio	90.2%	91.7%	89.1%	100%		
H. sapiens	47.3%	48.2%	49.1%	48.9%	100%	
M. musculus	42.9%	43.7%	45.6%	45.2%	51.4%	100%
